# The cell cycle of the planctomycete *Gemmata obscuriglobus *with respect to cell compartmentalization

**DOI:** 10.1186/1471-2121-10-4

**Published:** 2009-01-14

**Authors:** Kuo-Chang Lee, Rick I Webb, John A Fuerst

**Affiliations:** 1 School of Chemistry and Molecular Biosciences, University of Queensland, Brisbane, Queensland 4072, Australia; 2 Centre for Microscopy and Microanalysis, University of Queensland, Brisbane, Queensland 4072, Australia

## Abstract

**Background:**

*Gemmata obscuriglobus *is a distinctive member of the divergent phylum *Planctomycetes*, all known members of which are peptidoglycan-less bacteria with a shared compartmentalized cell structure and divide by a budding process. *G. obscuriglobus *in addition shares the unique feature that its nucleoid DNA is surrounded by an envelope consisting of two membranes forming an analogous structure to the membrane-bounded nucleoid of eukaryotes and therefore *G. obscuriglobus *forms a special model for cell biology. Draft genome data for *G. obscuriglobus *as well as complete genome sequences available so far for other planctomycetes indicate that the key bacterial cell division protein FtsZ is not present in these planctomycetes, so the cell division process in planctomycetes is of special comparative interest. The membrane-bounded nature of the nucleoid in *G. obscuriglobus *also suggests that special mechanisms for the distribution of this nuclear body to the bud and for distribution of chromosomal DNA might exist during division. It was therefore of interest to examine the cell division cycle in *G. obscuriglobus *and the process of nucleoid distribution and nuclear body formation during division in this planctomycete bacterium via light and electron microscopy.

**Results:**

Using phase contrast and fluorescence light microscopy, and transmission electron microscopy, the cell division cycle of *G. obscuriglobus *was determined. During the budding process, the bud was formed and developed in size from one point of the mother cell perimeter until separation. The matured daughter cell acted as a new mother cell and started its own budding cycle while the mother cell can itself initiate budding repeatedly. Fluorescence microscopy of DAPI-stained cells of *G. obscuriglobus *suggested that translocation of the nucleoid and formation of the bud did not occur at the same time. Confocal laser scanning light microscopy applied to cells stained for membranes as well as DNA confirmed the behaviour of the nucleoid and nucleoid envelope during cell division. Electron microscopy of cryosubstituted cells confirmed deductions from light microscopy concerning nucleoid presence in relation to the stage of budding, and showed that the nucleoid was observed to occur in both mother and bud cells only at later budding stages. It further suggested that nucleoid envelope formed only after the nucleoid was translocated into the bud, since envelopes only appeared in more mature buds, while naked nucleoids occurred in smaller buds. Nucleoid envelope appeared to originate from the intracytoplasmic membranes (ICM) of both mother cell and bud. There was always a connecting passage between mother cell and bud during the budding process until separation of the two cells. The division cycle of the nucleated planctomycete *G. obscuriglobus *appears to be a complex process in which chromosomal DNA is transported to the daughter cell bud after initial formation of the bud, and this can be performed repeatedly by a single mother cell.

**Conclusion:**

The division cycle of the nucleated planctomycete *G. obscuriglobus *is a complex process in which chromosomal nucleoid DNA is transported to the daughter cell bud after initial formation of a bud without nucleoid. The new bud nucleoid is initially naked and not surrounded by membrane, but eventually acquires a complete nucleoid envelope consisting of two closely apposed membranes as occurs in the mother cell. The membranes of the new nucleoid envelope surrounding the bud nucleoid are derived from intracytoplasmic membranes of both the mother cell and the bud. The cell division of *G. obscuriglobus *displays some unique features not known in cells of either prokaryotes or eukaryotes.

## Background

Members of phylum *Planctomycetes *of the domain Bacteria are distinctive budding peptidoglycan-less and compartmentalized bacteria from aquatic and soil habitats [1]. They are of increasing significance for evolution, molecular ecology, and cell biology [2]. The *Planctomycetes *comprise a distinct phylum of the domain Bacteria that forms a separate phylum on the basis of 16S rRNA analyses and have been proposed to be one of the deepest branching bacterial phyla [3] but have also been proposed to be related to the phyla *Verrucomicrobia *and *Chlamydiae *in the proposed PVC superphylum within the domain Bacteria [4].

Planctomycetes are increasingly significant for understanding of evolution, molecular ecology, and cell biology. They possess a number of distinctive phenotypic characteristics common to all members of the phylum. These include budding reproduction, peptidoglycan-less cell walls and a complex intracellular compartmentation. All planctomycetes share a cell plan in which the nucleoid DNA is enclosed by at least one membrane, the intracytoplasmic membrane (ICM) forming a major cell organelle, the pirellulosome [2,5,6]. The shared cell structure of planctomycetes also involves a ribosome-free region of the cytoplasm between cytoplasmic membrane and ICM termed the 'paryphoplasm'. Such features are optimally revealed under electron microscopy by the application of cryosubstitution and freeze-fracture. The genomic DNA of *Gemmata obscuriglobus *within the pirellulosome is further enclosed by two membranes forming an envelope surrounding a nuclear body organelle – the space between these membranes sometimes can be seen to be continuous with the paryphoplasm and the outer membrane of this nucleoid envelope may sometimes display continuity with the ICM [5]. The nuclear body contains most if not all of the cell DNA in the form of the fibrillar nucleoid, as well as ribosome-like particles which also occur in the cytoplasm external the nuclear body.

Models for the cell division cycle in bacteria have been based largely on either species displaying symmetric binary fission or on *Caulobacte*r displaying asymmetric division correlated with a prosthecate stalk [7-10]. Other asymmetric models have also been described in preliminary terms [11]. In all of these cases, the nucleoid appears to be free in the cytoplasm without membrane boundaries. Segregation of chromosomes bounded by a membrane envelope as in *G. obscuriglobus *may pose problems concerning whether DNA attachment to cytoplasmic membrane either directly or via DNA-binding membrane proteins could apply, and whether the membranes of the nuclear body disassemble during division or are retained throughout division in an analogous way to those of yeast nuclei during 'closed mitosis' [12]. Concerning cell division itself, one of the remarkable features of planctomycetes including *Gemmata *strains, although based only on draft genomes is the absence of the cell division protein FtsZ otherwise conserved among the domain Bacteria other than the phylum *Chlamydiae *[13-15]. Other mechanisms may apply to planctomycetes, and this may be reflected in the mode of cell division and chromosome segregation occurring in *G. obscuriglobus*.

The cell division cycle of *G obscuriglobus *is important to examine for several reasons. The way in which its membrane-bounded nucleoid is distributed during cell division is not known and clearly relates to the problem of how chromosomes are segregated in this compartmentalized organism. Secondly, since FtsZ seems absent in planctomycetes for which genome sequence data exists (including draft genome for 2 *Gemmata *strains); the cell division cycle must present features distinct from other bacteria. Thirdly, comparison of the division cycle of planctomycetes with other budding bacteria in different phyla may reveal either a unity or a diversity of mechanisms for what appears to be a similar process superficially.

Other budding bacteria are known in the alpha-proteobacteria of the phylum *Proteobacteria*, and such dimorphic prosthecate bacteria also display asymmetric cell division, but correlated with the presence of a prostheca, an extension of the cell cytoplasm and wall [16].

In this study we have examined the cell cycle of *Gemmata obscuriglobus *using phase contrast and fluorescence light microscopy including confocal laser scanning microscopy as well as electron microscopy.

## Results

### Time-lapse experiment

**Figure 1:**
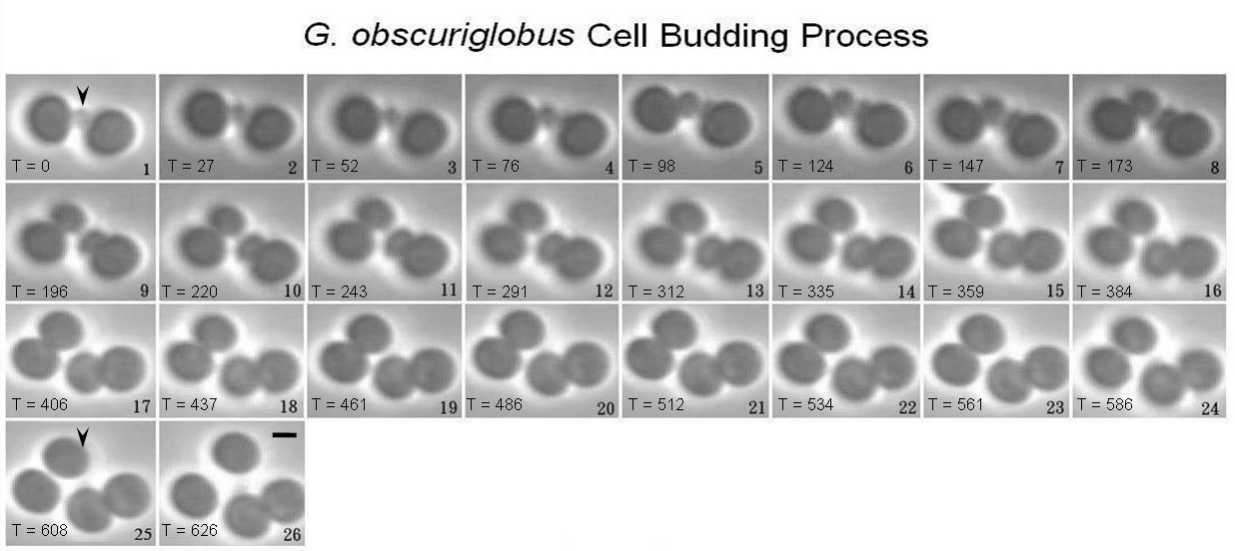
**Time-lapse phase contrast micrographs showing *G. obscuriglobus *budding cell division**. Bud initiates from the polar end of the mother cell, grows until it reaches the same size as the mother cell and finally separates from the mother cell. The full budding process takes over 10 hours and approximately 12 hours. Arrowhead indicates the bud at the initiation (frame 1) and after separation (frame 25). *Unit of the time in each frame is minutes*. Bar – 1 μm.

*Gemmata obscuriglobus *budding cells growing on an M1 agar medium slab were examined and micrographs were captured from time-lapse phase contrast microscopy. The budding cell division occupied approximately 12 hours from initial bud formation until separation from the mother cell (Fig. 1). Observations showed that the bud formed at one point of the mother cell perimeter and developed until it reached the same cell size as the mother cell, finally separating from the mother cell. It was noted that there was a period lasting approximately 4 hours between the time when the bud had developed to a similar size as the mother cell until separation of the two cells. The matured daughter cell (bud) can act as a new mother cell and start its own budding cycle after separation from the mother cell while the mother cell can initiate a new budding cycle repeatedly.

A mother cell took approximately 2–4 hours to initiate a new budding cycle after separation of the first mature daughter cell while the matured daughter needed a longer time of 3–5.5 hours to start its own budding cycle. There were some cases where a new bud formed between the mother cell and the mature daughter while they seemed to be still in close contact, which can be described as intercalary budding (similar to that occurring in another planctomycete *Isophaera pallida*) [17]. It was also seen that new buds were repeatedly formed from the same position on the mother cell surface on which the previous bud was formed, thus suggesting there is a reproductive pole on the mother cell. This also applies to the matured daughter cell from which a new bud is always formed from the same pole.

### Fluorescence light microscopy

**Figure 2:**
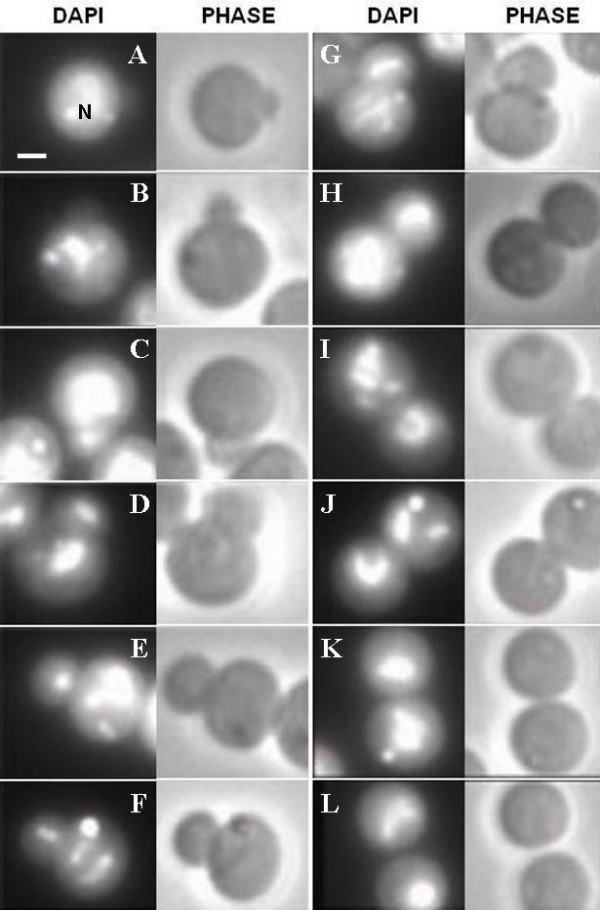
**Fluorescence and phase contrast micrographs of individual *G. obscuriglobus *budding cells**. Each pair of micrographs (DAPI and PHASE) represents a different budding stage seen after staining with DAPI and visualizing via fluorescence and phase contrast microscopy, and each letter A-L refers to a paired micrograph (DAPI-stained on the left and PHASE microscopy on the right) of a different cell in each case. Nucleoid (N) DNA is stained with DAPI. Pair A represents the earliest budding stage while pair L represents the final stage. Bar – 1 μm.

*Gemmata obscuriglobus *budding cells from an asynchronous culture were DNA-stained with DAPI and individual budding cells within this population representing different stages of budding were observed. The spectrum of cell types in micrographs of *G. obscuriglobus *DAPI-stained cells suggested that formation of the bud and translocation of the nucleoid into the bud did not occur at the same time. Budding cells at an early stage of the budding division cycle clearly show no sign of DNA inside their buds (see Fig. 2A and 2B). Presence of DNA was observed to occur in both mother and bud cells only at later budding stages (Fig. 2C and onwards). Since this was not a time-lapse result, we could not interpret how long after the initiation of the budding process that the DNA is translocated into the bud. However, it seems clearly that new bud formation can occur without simultaneous transfer of the nucleoid to the bud, so that there is a disconnection between bud formation and chromosome segregation into the bud [18].

### Confocal laser scanning microscopy (CLSM)

**Figure 3:**
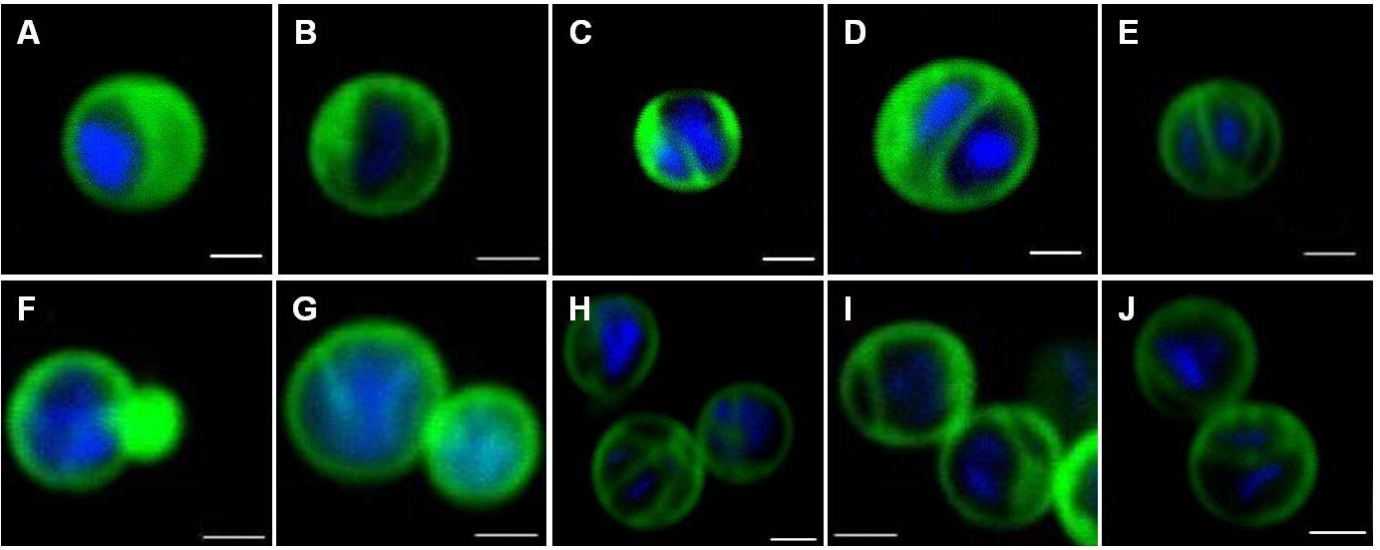
**Confocal laser scanning micrographs of individual *G. obscuriglobus *budding cells stained with DAPI and DiOC_6_**. Each micrograph (A-J) represents either example of different appearances of cells before budding or different stages of budding. Frames (A-E) represent cells without buds (interpreted as cells seen before budding), frame (F) represents the earliest budding stage, frames (G-I) later stages of more mature buds and frame (J) the final stage. Blue – DAPI, Green – DiOC_6_, Bar – 1 μm.

Double staining of populations of *G. obscuriglobus *budding cells in asynchronous culture with DAPI and DiOC_6 _dyes combined with viewing by CLSM yielded results consistent with those from non-confocal fluorescence light microscopy (Fig. 3). It was noted that single cells without buds (interpreted as cells before bud initiation) can either carry one nucleoid or two nucleoids separated by membranes (Fig. 3A–E). Cells in the early budding stage showed a nucleoid-containing mother cell and a small bud without the presence of a nucleoid (Fig. 3F). In cells representing later budding stages, both the mother cell and the bud were found to contain a nucleoid and DiOC_6_-staining internal membranes surrounding the nucleoid interpreted as nucleoid envelope and the ICM (Fig. 3G–J).

### Transmission electron microscopy

**Figure 4:**
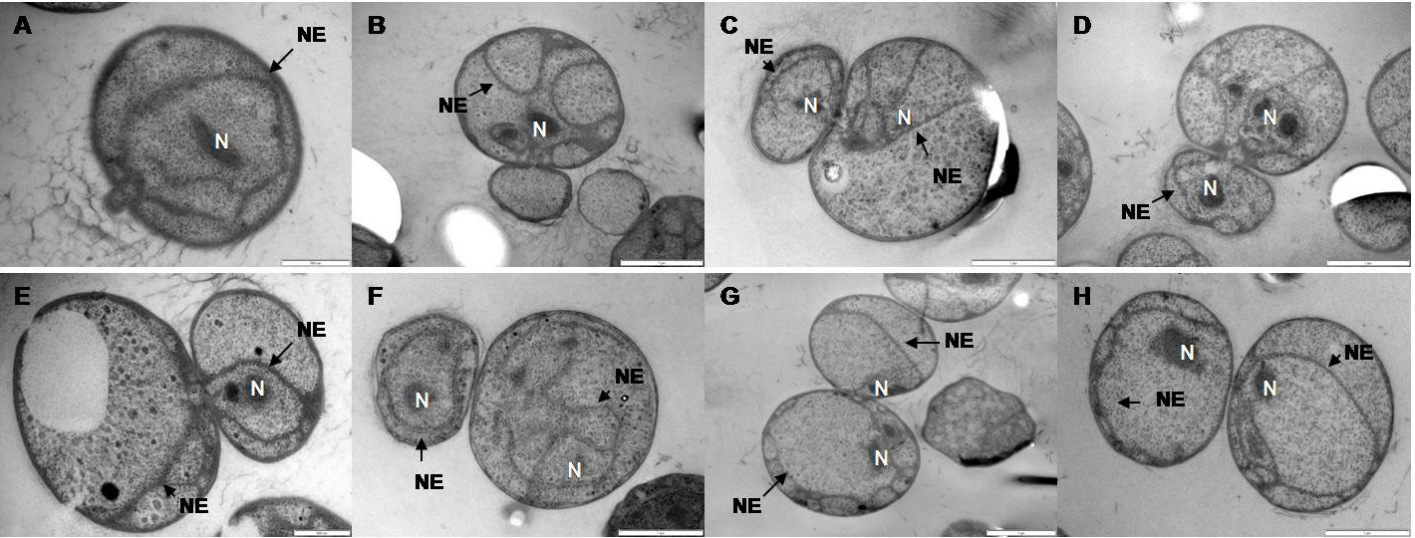
**Transmission electron micrographs of high-pressure frozen, cryosubstituted and Epon-embedded *G. obscuriglobus *budding cells**. Each micrograph represents a different budding stage. The bud initiates from the polar end of the mother cell containing a nucleoid (N) surrounded by a nucleoid envelope (NE) (A) and the bud increases in size without the presence of either the nucleoid or the nucleoid envelope (B). As the bud develops, the nucleoid is translocated into the bud and the nucleoid envelope starts to develop (C, D and E) until forming a fully enclosed nuclear body (F). The bud continues developing until reaching a similar size to that of the mother cell (G), and the matured bud finally separates from the mother cell (H).Bar 500 nm (images A and E), 1 μm (images B, C, D, F, G and H).

Populations of *G. obscuriglobus *budding cells in asynchronous culture were high-pressure frozen, cryosubstituted and thin-sectioned and examined by transmission electron microscopy. Individual *G. obscuriglobus *cells likely to represent different stages of the budding division cycle were selected. Cells in all stages clearly displayed the internal compartmentalization characteristic of planctomycetes including the ICM-defined pirellulosome containing the double-membrane bounded nucleoid and the paryphoplasm region between the ICM and CM. Fig. 4 demonstrates the fate of the nucleoid and the nuclear membrane in the bud relative to bud development throughout the budding division cycle. Membrane-enclosed nucleoids were always present in the mother cell throughout the budding division cycle. Both nucleoid and nucleoid envelope were found to be absent in the small initial-stage buds, which do possess a paryphoplasm and ICM. A nucleoid was observed only within buds representing larger more advanced budding stages. When the nucleoid is first seen in the bud it always appears in condensed form.

**Figure 5:**
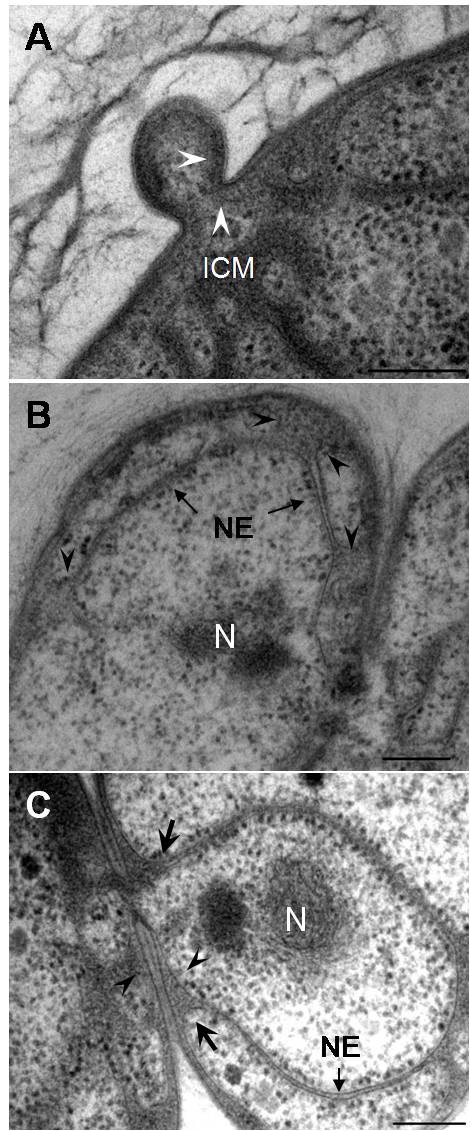
**Transmission electron micrographs of high-pressure frozen, cryosubstituted and Epon-embedded *G. obscuriglobus *at different budding stages**. A. The ICM of the bud is continuous (arrowheads) with the mother cell ICM through the bud neck during an early budding stage. No nucleoid is visible in the bud at this stage. B. Condensed nucleoid is visible in this more matured bud and double membrane nucleoid envelope (NE) is visible in some places. The nucleoid envelope does not yet completely surround the nucleoid. The arrows indicate several regions at which ICM of the bud is continuous with the outer membrane of the nucleoid envelope. C. Budding cell displaying the origin of the double-membrane nucleoid envelope (NE) surrounding the nucleoid (N). The mother cell ICM displays continuity (arrowheads) to the inner membrane of the nucleoid envelope, and the bud ICM shows continuity (arrows) to the outer membrane of the nucleoid envelope. Bar – 200 nm.

Internal membranes within the cytoplasm which can be interpreted as nucleoid envelopes or precursors to nucleoid envelopes (on the criterion of two closely apposed membranes) were only observed in buds with nucleoid presence. We conclude from this that formation of the nucleoid envelope occurs only after the nucleoid has been translocated into the bud. Each membrane of the double-membraned nucleoid envelope appeared to have originated from ICMs, one membrane from the ICM of the mother cell and the other from that of the bud. As judged from membrane continuity, the bud's ICM seems to originate initially from the ICM of the mother cell (Fig. 5A). There was always a connecting neck passage between the mother cell and the bud throughout the budding process where continuity of the cell wall, cytoplasmic membrane and ICM were observed. In a more matured bud (Fig. 5B), condensed nucleoid is visible within the cell cytoplasm and there are regions of double membrane also present similar to mature nucleoid envelope but they do not at this stage form a continuous envelope surrounding the nucleoid. Nucleoids in buds not fully enclosed in nucleoid envelope membranes in this study may conceivably prove to be enclosed if tomography or serial sectioning were performed on such buds.

In several regions, the ICM of the bud appears to be continuous with the outer membrane of the putative nucleoid envelope. Regions of paryphoplasm separate the membranes where ICM is continuous with the putative outer nuclear membrane. Thus several vesicles are effectively formed by a continuous membrane representing both the ICM extensions and the putative outer nuclear membrane. These vesicles contain cytoplasm with ribosome-like particles. In a more matured stage of budding where nucleoid envelope almost complete surrounds the nucleoid (Fig. 5C), the ICM of the bud is continuous with the outer membrane of the bud nucleoid envelope, while the inner membrane of this nucleoid envelope seems to be continuous with the ICM of the mother cell, so presumably is formed from that ICM. This interpretation is also consistent with the appearance of the origin of the inner membrane of putative nucleoid envelope segments in Fig. 5B. Both inner and outer nucleoid envelope membranes thus appear to form from ICMs but parental ICM in one case and daughter ICM in the other. In buds with nucleoid envelopes, ribosomes aggregate and form a line at both sides of the nucleoid envelope, that is along both the inner and the outer envelope membranes. Such ribosome arrangement along the nucleoid envelope membranes could also be seen in the mother cell. Putative nucleoid envelope apparently in the process of formation seen in less matured buds also appears to associate with ribosome-like particles. The association of the ribosome-like particles with nucleoid envelope appears to be a characteristic phenomenon of the nucleoid envelope of *G. obscuriglobus *when prepared by freeze substitution. It may form a useful marker of nucleoid envelope in such cells.

## Discussion

**Figure 6:**
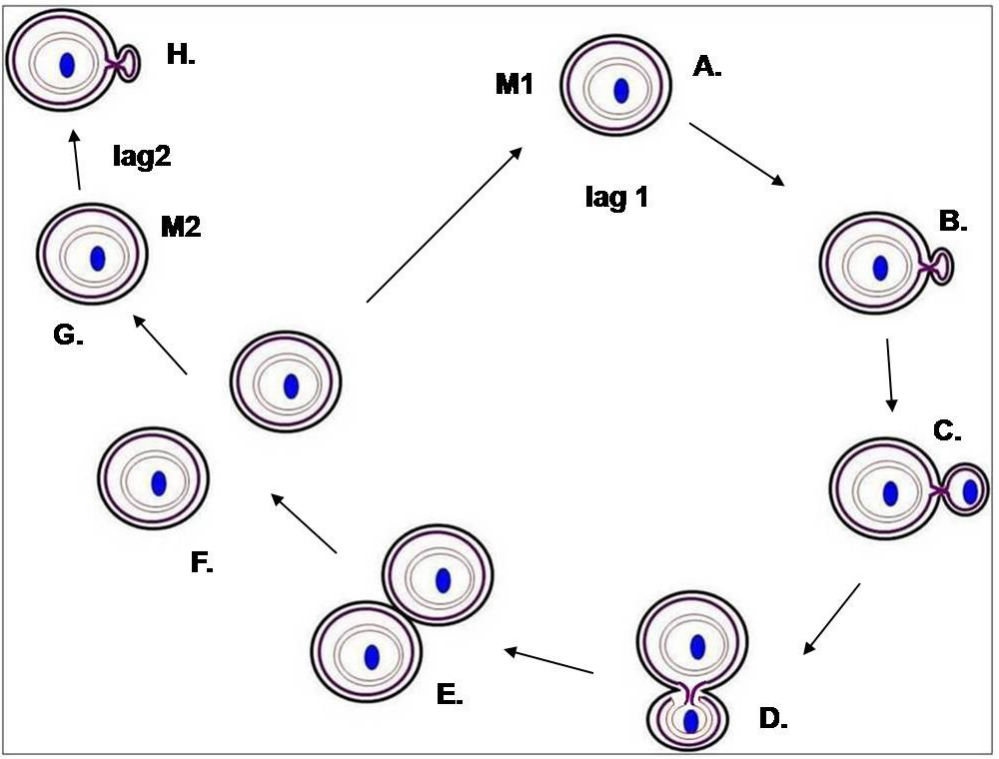
***Gemmata obscuriglobus *cell budding cycle**. In (A) and (B), a bud initiates from one point of the mother cell M1 enclosing a nucleoid (blue) surrounded by a double-membrane nucleoid envelope (grey). The mother cell ICM (dark purple) is continuous with that of the bud (also dark purple). In (C), the naked nucleoid is translocated into the bud at some early budding stage. In (D), the bud nucleoid is surrounded by two membranes where an inner membrane (light purple) continuous with mother cell ICM, and an outer membrane (also light purple) shows continuity with the bud ICM. In (E), the bud nucleoid is completely surrounded by the two closely apposed membranes where membrane fusion and pinching off has resulted in a double-membrane nucleoid envelope completely separated from ICM membranes. (E) is the end-point of a possible model mechanism where the bud reached similar cell size as M1. (F) shows the separation of the mother cell and the matured bud. In (G) and (H), the mother cell M1 can initiate the next budding cycle after a 2–4 hour lag (lag 1) while the matured bud M2 can begin its first budding cycle after a 3–5.5 hour lag (lag2).

Little is known about the cell division cycle of the compartmentalized planctomycetes, especially how membrane-bounded compartments such as those enclosing the nucleoid are transferred into the daughter cells. Results of phase contrast and fluorescence light microscopy combined with electron microscopy of thin-sectioned cells prepared by high pressure freezing/cryosubstitution can be used to derive a model for the cell cycle of *Gemmata obscuriglobus*. The *G. obscuriglobus *cell cycle is summarized in Fig. 6. In this model a mother cell forms a small bud with a narrow neck relative to mother cell diameter, and this bud gradually enlarges until it is similar in size to the mother cell, a stage which then lasts for a time considerably longer than other stages of cell division. Both the mother cell and the finally released bud are capable of further cell division – the bud can only start this after a lag period which is much longer than the lag observed for the second mother cell budding. There appears to be a distinct reproductive pole, since division seems to occur repeatedly at the same pole. A new bud is formed at the same pole position of the mother cell where the previous bud was formed, matured and separated from the mother cell.

During cell division, the earliest visible bud stage does not possess DNA via DAPI-staining and a fibrillar nucleoid is not observed in such sectioned buds. Electron microscopy of cells prepared by high pressure freezing/cryosubstitution shows that a nucleoid is initially visible in the bud before a complete nucleoid envelope is formed, suggesting that a nucleoid-containing nuclear body is not transferred intact as a membrane-enclosed structure to the bud. The origin of the new nucleoid envelope found in the bud from both ICM of mother cell and ICM of the bud implies that the *G. obscuriglobus *nucleoid envelope does not form from mother cell nucleoid envelope, but that it is formed by de novo membrane synthesis as an extension of ICM membrane – this may happen at each cell division. The nucleoid thus appears to be transferred to the bud at first in a naked form, and nucleoid envelope is synthesized around it as extensions of ICM membranes from both mother cell and bud. In Fig. 6 describing one possible model for the cell cycle of *G. obscuriglobus*, critical stages are those between Fig. 6B and Fig. 6C, where the nucleoid appears in the bud (which initially shows no nucleoid) and then becomes surrounded by two closely apposed membranes to form a new nucleoid envelope for the new bud nucleoid. We have no evidence relating to exactly how the naked nucleoid enters the bud, or whether this involves (as it might) the opening of the mother cell nucleoid envelope membranes to allow passage of a new nucleoid to the bud, so we have not shown such a possible stage in Fig. 6. It should be noted also that Fig. 6D illustrates a model consistent with the appearance of the cell in Fig. 5C, in that the nucleoid (blue) of the bud is now surrounded by two membranes an inner membrane (in light purple) continuous with mother cell ICM (dark purple), and an outer membrane (also in light purple) which shows continuity with the ICM of the bud (in dark purple) at two regions on either side of the bud neck. A possible intermediate stage between Fig. 6C and 6D is suggested by the appearance of the bud in Fig. 4C and 4D and Fig. 5B, where the nucleoid is only partially surrounded by membranes derived from mother ICM and bud ICM. In Fig. 5B, there appear to be multiple regions at which ICM of the bud is continuous with the outer membrane of the nucleoid envelope, so the outer nucleoid envelope membrane at least may not necessarily be formed at a single or only two points of continuity with the bud cell's ICM, as might be implied by the late stage in Fig. 5C and Fig. 6D where the nucleoid is almost completely surrounded by the mature nucleoid envelope consisting of two apposed membranes, one derived from the mother cell's ICM and one derived from the bud cell's ICM.

There thus appears to be an intimate relation between the ICM and nucleoid envelope in *G. obscuriglobus*. This is consistent with the continuity noted previously between the outer nucleoid envelope membrane and the ICM in cryosubstituted *G. obscuriglobus *cells [5]. The distribution of the nucleus and nucleoid in *G. obscuriglobus *is thus not analogous to closed mitosis in some eukaryotes such as yeasts where the nucleus and its envelope is distributed to the daughter cell intact [19], nor to open mitosis of other eukaryotes such as animal cells where the nucleoid envelope breaks down during mitosis and reassembles afterwards [12,20,21]. However, there is now evidence that even in open mitosis, newly assembles nuclear membrane actually derives from existing endoplasmic reticulum membrane [22]. In the metazoan *Xenopus*, the new nucleoid envelope (NE) formation is initiated by endoplasmic reticulum (ER) tubule-end binding and subsequent tethering of the ER network on chromatin. Chromatin was shown to play an active role in reshaping of the ER during NE formation; therefore the process of nuclear membrane formation in *G. obscuriglobus*, which coincides with the presence of the nucleoid in the buds of *G. obscuriglobus*, may be analogous in some ways. The occurrence of an early bud without a nucleoid followed by migration of nucleus into the new bud is analogous to the order of nucleoid appearance occurring in budding yeast [23], but presumably without an M phase mitotic segregation of chromosomes into the bud. Overall, the cell division of *G. obscuriglobus *displays some unique features not known in cells of either prokaryotes or eukaryotes. For example, a nucleoid envelope forms in the daughter cell around the nucleoid (not occurring in other prokaryotes) and there does not seem to be a process of eukaryote-like mitosis in these bacteria. Such a process would be expected to involve in open mitosis a disassembly of mother cell nucleoid envelope and in closed mitosis no stage at which a naked nucleoid (without nucleoid envelope) occurs and in both forms of mitosis, mitotic spindles composed of microtubules. Unlike any form of mitosis we can identify in eukaryotes, although the mother cell in dividing *G. obscuriglobus *retains enveloped nucleoids, the nucleoid in the bud is initially naked or only enveloped by ICM of the bud, and new nucleoid envelope is then apparently derived from existing intracellular membranes of both mother and daughter cells. The new bud outer nucleoid envelope membrane can easily be conceived to form by a formation of vesicle blebbing of the daughter bud cell ICM membrane since we have evidence for multiple vesicles forming the outer nucleoid envelope of the bud and still connected to the ICM. Continuity of the new inner nucleoid membrane with the mother cell ICM is seen and more difficult to explain mechanistically; the mother cell nucleoid envelope does not appear to be directly involved in formation of the bud nucleoid envelope but it may be that it must open at some point, perhaps the bud neck, in order for the nucleoid to pass to the bud – this stage is not clearly captured in our micrographs however so has not been assumed in our model for this process. In an alternative model, it may also be that the new bud nucleoid envelope membranes form via de novo membrane synthesis but that the ICM membranes act as seed points. The existing data do not discriminate between such models involving extension of existing membrane or formation of new membrane on a framework of existing membranes.

Sectioned *G. obscuriglobus *budding cells prepared via high-pressure freezing/cryosubstitution display nucleoids which always appear in condensed form, whether in the mother cell or the bud. This contrasts with the normal case in prokaryotic cells such as *E. coli *where DNA does not condense during cell division. It suggests that either the nucleoid remains condensed during division or is recondensed after a strand unfolding if that occurs during division. It may be most likely that a condensed nucleoid is transferred to the bud since there is a very early stage of budding without DAPI-stainable DNA, and the next stage distinguishable has a condensed nucleoid as seen via TEM of thin-sectioned high pressure frozen/cryosubstituted cells.

In buds with nucleoids and nucleoid envelopes, ribosomes aggregate and are arranged linearly at both sides of the nucleoid envelope, that is, along both the inner and the outer envelope membranes. Such ribosome arrangement along the nucleoid envelope membranes could also be seen in the mother cell and might be a phenomenon of the nucleoid envelope of *G. obscuriglobus *preserved when prepared by high-pressure freezing. It may form a useful marker of nucleoid envelope in such cells. Such arrangement implies that co-translational protein secretion might occur across nuclear body membranes at some stages e.g. in a newly formed bud, making them analogous to eukaryote ER in some ways.

The only other planctomycete for which the cell division cycle has been described is a freshwater strain once classified as Morphotype IV of the '*Blastocaulis-Planctomyces' *group, ICPB 4232, closely related to ATCC35122 and therefore to *Pirellula staleyi *[24], and in that strain a motile swarmer daughter develops at one pole of a non-motile daughter cell [25]. We have examined negatively stained *Gemmata obscuriglobus *cells and single cells of mother cell size possess a tuft of flagella, and this view that mother cells before or between budding are motile is also confirmed in phase contrast microscopy of wet mount, where most or all cells are motile. In ICPB 4232, as much as 30 hours were required for the swarmer to initiate a new budding cycle after becoming sessile, while *G. obscuriglobus *buds also exhibited a lag in bud formation but this lag was only 3–5.5 hours. In ICPB 4232, budding formation and maturation to separation occupied 3 hours under the conditions used, while in *G*. obscuriglobus budding cell division occupied approximately 12 hours from initial bud formation until separation from the mother cell. In ICPB 4232, there is a 'resting phase' lag of 7–9 hours before a new bud forms, while in *G. obscuriglobus *there is also a lag in new mother cell budding, of 2–4 hours. Of course such times may vary in any case with different culture media and strain growth rate. The structure of the cell division cycle may be similar in these two planctomycetes, but *G. obscuriglobus *may not differentiate into a distinct swarmer stage.

The closest analogs to the *G. obscuriglobus *mode of cell division within the Bacteria are the budding prosthecate bacteria *Hyphomicrobium *and *Hyphomonas*. In *Hyphomicrobium *sp. strain B522, nucleoids are absent in very young buds [26] and this is similar to the situation observed in *G. obscuriglobus*. In the prosthecate bacterium *Hyphomonas*, the nucleoid DNA is partitioned to the swarmer cell and transferred to the swarmer cell through a prosthecate hypha via 'pseudovesicles' surrounded by cytoplasmic membrane and containing ribosomes as well as DNA [16]. This does not appear to occur in *G. obscuriglobus*, but the stage where actual nucleoid transfer occurs has not been captured, perhaps because this is relatively rapid and thus cells displaying it occur in very low numbers in thin sections.

The phylum *Planctomycetes *to which *Gemmata obscuriglobus *belongs has been proposed to be a member of the PVC superphylum comprising at least the phyla *Verrucomicrobia *and *Chlamydiae *as well as the *Planctomycetes *[4,27,28]. Members of the phylum *Chlamydiae *display a distinctive life cycle during the infection of eukaryote cells by these pathogens, including a reticulate body stage capable of division by binary fission and an infective elementary body cell stage [29]. The latter displays condensed nucleoids analogous to those of *G. obscuriglobus*, but this condensation is released in the reticulate dividing stage, unlike the situation in dividing *G. obscuriglobus*. There is also as yet no evidence of cell compartmentalization in chlamydiae.

## Conclusion

The division cycle of the nucleated planctomycete *G. obscuriglobus *is a complex process in which naked chromosomal DNA is transported to the daughter cell bud after initial formation of the bud. DNA is transported into the bud in the later stages of budding and then surrounded by a nucleoid envelope forming a new nuclear body, completing the compartmentalization of the new bud. Budding division can be performed repeatedly by a single mother cell. These results can form the basis for further progress at the molecular level to elucidate the developmental biology of this planctomycete model for cell division in a peptidoglycan-less and compartmentalized bacterium, for example to investigate which proteins may be associated with the chromosome of the nucleoid during segregation into the bud and whether histone-like proteins are involved in the condensation of nucleoid in a similar manner to that in the chlamydial elementary body nucleoid, as well as how intracytoplasmic membranes are organized to form the nucleoid envelope of the new bud, possibly via interactions with the newly transferred nucleoid.

## Methods

### Bacteria and culture conditions

*G. obscuriglobus *type strain, UQM 2246 (University of Queensland Department of Microbiology Culture Collection strain 2246) was grown on M1 medium [30] incubated aerobically at 28°C for 2 to 7 days.

### Phase contrast microscopy and time-course experiment

4-day *Gemmata obscuriglobus *cells grown on M1 medium were harvested into sterile-filtered Milli-Q grade deionised water and 5 μl of suspension was deposited onto a M1 medium block sit on a glass slide as in the agarose slab method described below. The specimen was transferred to a Petri dish and incubated aerobically at 28°C overnight. Before observation, a cover slip was placed onto the medium block seeded with *Gemmata obscuriglobus *cells. Samples were analyzed by direct microscopic observation through 100× lens objective. Cells on one field were photographed every 28 minutes for 8 to 10 hours. Images were captured with a Zeiss Axioplan 2 universal microscope in conjunction with Zeiss KS 200 v. 3.0 software and a Variocam Digital Video black and white camera.

### Cell staining and wide-field fluorescence microscopy

2- to 3-day *Gemmata obscuriglobus *cells grown on M1 agar plates were harvested into sterile-filtered Milli-Q grade deionised water. After centrifuging, the pellet was resuspended in dye solution for 5 min in the dark before pipetting 5 μl onto an agarose-slab on a glass microscope slide, allowed to soak in and a cover slip placed on top. The dye used was DAPI (4'-6-diamidino-2-phenylindole) with final concentration of 3.3 μg/ml. Specimens were viewed using a Zeiss Axioplan 2 universal microscope in conjunction with Zeiss KS 200 v. 3.0 software and a Variocam Digital Video black and white camera, and using a 100× fluorescence objective. Images were captured using excitation-emission filter block of 365 nm. Interpretations of the DNA distribution at different cell stages were made from at least 100 cells.

### Confocal laser scanning microscopy

2- to 3-day *Gemmata obscuriglobus *cells grown on M1 agar plates were harvested into sterile-filtered Milli-Q grade deionised water. After centrifuging, the pellet was resuspended in dye solution for 5 min in dark. The dye solution contained DAPI and DiOC_6 _with both final concentrations of 3.3 μg/ml. Specimen were then washed and resuspended in 1% warm agarose solution. 10 μl of specimen were applied onto a glass slide and placed on a cover slip. Specimens were viewed using Zeiss LSM 510 Meta confocal laser scanning microscope equipped with LSM 510 Image Software and using a 100× fluorescence objective. Multi-track of appropriate excitation-emission filter blocks (365 nm excitation of DAPI, 450–490 nm excitation of DiOC_6_) were used and images of the emissions from both stains were merged automatically. Final images were brightness and contrast adjusted using LSM Image Browser.

### High-pressure freezing and transmission electron microscopy

Cultures that were to be examined via electron microscopy were high-pressure frozen with liquid nitrogen using the BalTec HPM-010 or the Leica EMPACT 2 high-pressure freezer. The frozen samples were kept and stored in a 2 ml tube containing liquid nitrogen before cryosubstitution.

The frozen sample was transferred to an eppendorf tube containing 2% osmium tetroxide in acetone and cryosubstituted in Leica AFS where the sample is warmed from -160°C to -85°C over 1.9 hrs (rate 40°C/hr); -85°C for 36 hrs; -85°C to 20°C over 11 hrs (4°C/hr). The high-pressure frozen and cryosubstituted samples were then processed into Epon resin and thin-sectioned using the Leica Ultracut Ultramicrotome UC6. The thin sections were placed onto a formvar-coated copper grid and stained with 5% uranyl acetate in 50% ethanol and lead citrate.

All high-pressure frozen/cryosubstituted sections were viewed using a JEOL 1010 transmission electron microscope operated at 80 kV. Images were captured using a SIS Megaview III digital camera. The resulting files were annotated for final image production using Adobe Photoshop CS version 8.0.

## Authors' contributions

K-CL cultured and prepared cells for and performed phase contrast time-lapse microscopy, fluorescence microscopy, high-pressure freezing and electron microscopy. RIW assisted K-CL with expert knowledge of high-pressure freezing cell preparation. K-CL and JAF wrote the manuscript and RIW contributed to drafting the manuscript. JAF conceived of the study, participated in its design and coordination and helped to write the manuscript. All authors read and approved the final manuscript.
